# The role of *SND2* in the regulation of *Arabidopsis* fibre secondary cell wall formation

**DOI:** 10.1186/1753-6561-5-S7-P114

**Published:** 2011-09-13

**Authors:** Steven Hussey, Eshchar Mizrachi, David Berger, Alexander Myburg

**Affiliations:** 1 Department of Genetics, Forestry and Agricultural Biotechnology Institute (FABI), University of Pretoria, Pretoria, South Africa; 2 Department of Plant Science, Forestry and Agricultural Biotechnology Institute (FABI), University of Pretoria, South Africa

## Background

Transcription factors (TFs) play important roles in the regulation of secondary cell wall (SCW) biosynthesis in herbaceous and woody plants. In *Arabidopsis*, the onset of SCW deposition is initiated by a nexus of NAC, MYB, homeodomain and several other families of TFs, which function in a transcriptional network regulating SCW biosynthetic genes. NAC family members SND1/NST1 and VND6/VND7 have been identified as functionally redundant master regulators of SCW formation in fibres and vessels, respectively [1,2]. *Arabidopsis* plants overexpressing *SND2*, an indirect target of fibre master regulator SND1, exhibited increased SCW thickness in inflorescence stem fibres, whilst dominant repression lines exhibited a decrease in fibre SCW thickness associated with a reduction in glucose and xylose cell wall sugar content [3]. The ability of *SND2* to transactivate the *CesA8* promoter [3] suggested that *SND2* may regulate cellulose biosynthetic genes during fibre SCW formation. The evaluation of this hypothesis necessitates the identification of all downstream genes potentially regulated by *SND2*, and the analysis of SCW chemistry and morphology in overexpression lines. The aim of this ongoing study is to further elucidate the role of *SND2* in fibre SCW formation through microarray analysis of overexpression lines and by independent confirmation of the effect of *SND2* overexpression on fibre SCW chemistry and morphology in *Arabidopsis* plants.

## Methods

We generated *2x35S::SND2 Arabidopsis thaliana* Col-0 plants, screened them for *CesA8* upregulation and phenotypically assessed several homozygous (T4) transgenic lines. Inflorescence stem fibre SCW thickness was measured from light and scanning electron micrographs, and the cell wall monosaccharide and Klason lignin composition of stems was determined relative to the wild type. We performed microarray analysis of the inflorescence stem transcriptomes of wild type and transgenic *Arabidopsis* plants using the Agilent 4x44k transcriptome array and confirmed the expression profiles of differentially expressed genes with RT-qPCR.

## Results

Transgenic *Arabidopsis* (T4) lines showed no significant external phenotype, and we were unable to reproduce the increased fibre SCW thickness phenotype reported by Zhong *et al.*[3]. We identified a single homozygous line with *CesA8* upregulation and moderate *SND2* overexpression. Whole-transcriptome analysis of the line revealed the upregulation of several TFs and genes associated with SCW biosynthesis, which were reproducibly upregulated in an independent trial. We additionally observed possible ectopic artifacts and gene dosage effects associated with excessive constitutive expression of the *SND2* gene*.* Chemical analysis revealed only minor changes in SCW monosaccharides, despite the upregulation of SCW biosynthetic genes.

## Conclusions

Our results implicate *SND2* in the regulation of cellulosic and non-cellulosic components of fibre cell walls, and we provide a model for the position of *SND2* in the transcriptional network regulating fibre SCW formation.

## References

[B1] Kubo M Udagawa M Nishikubo N Horiguchi G Yamaguchi M Ito J Mimura T Fukuda H Demura T Transcription switches for protoxylem and metaxylem vessel formation Genes and Development 2005 19 1855 1860 10.1101/gad.1331305 16103214 PMC1186185

[B2] Zhong R Richardson EA Ye Z-H Two NAC domain transcription factors, SND1 and NST1, function redundantly in regulation of secondary wall synthesis in fibers of *Arabidopsis* Planta 2007 225 1603 1611 10.1007/s00425-007-0498-y 17333250

[B3] Zhong R Lee C Zhou J McCarthy RL Ye Z-H A Battery of Transcription Factors Involved in the Regulation of Secondary Cell Wall Biosynthesis in *Arabidopsis* The Plant Cell 2008 20 2763 2782 10.1105/tpc.108.061325 18952777 PMC2590737

